# Intensive Blood Pressure Control After Endovascular Thrombectomy for Acute Ischemic Stroke: a Systematic Review and Meta-Analysis

**DOI:** 10.1007/s00062-024-01391-6

**Published:** 2024-03-07

**Authors:** Mohamed Abuelazm, Yehya Khlidj, Ahmed A. Ibrahim, Abdelrahman Mahmoud, Ahmed Mazen Amin, Ibrahim Gowaily, Ubaid Khan, Basel Abdelazeem, James Robert Brašić

**Affiliations:** 1 Faculty of Medicine, Tanta University, Tanta, Egypt; 2 Faculty of Medicine, University of Algiers, Algiers, Algeria; 3 Faculty of Medicine, Menoufia University, Menoufia, Egypt; 4 Faculty of Medicine, Minia University, Minia, Egypt; 5 Faculty of Medicine, Mansoura University, Mansoura, Egypt; 6 Faculty of Medicine, King Edward Medical University, Lahore, Pakistan; 7 Department of Cardiology, West Virginia University, West Virginia, USA; 8 Section of High-Resolution Brain Positron Emission Tomography Imaging, Division of Nuclear Medicine and Molecular Imaging, The Russell H. Morgan Department of Radiology and Radiological Science, The Johns Hopkins University School of Medicine, Baltimore, MD USA; 9 Department of Behavioral Health, New York City Health and Hospitals/Bellevue, New York, NY USA; 10 Department of Psychiatry, New York University Grossman School of Medicine, New York University Langone Health, New York, NY USA

**Keywords:** Stroke, Cardiovascular disease, Clinical trial, Function recovery, Intracranial hemorrhage, Meta-analysis

## Abstract

**Background and Purpose:**

Optimal clinical outcome with successful recanalization from endovascular thrombectomy (EVT) requires optimal blood pressure (BP) management. We aimed to evaluate the efficacy and safety of the intensive BP target (< 140 mm Hg) versus the standard BP target (< 180 mm Hg) after EVT for acute ischemic stroke.

**Methods:**

We conducted a systematic review and meta-analysis synthesizing evidence from randomized controlled trials (RCTs) obtained from PubMed, Embase Cochrane, Scopus, and WOS until September 7th, 2023. We used the fixed-effect model to report dichotomous outcomes using risk ratio (RR) and continuous outcomes using mean difference (MD), with a 95% confidence interval (CI). PROSPERO ID: CRD42023463206.

**Results:**

We included four RCTs with 1559 patients. There was no difference between intensive BP and standard BP targets regarding the National Institutes of Health Stroke Scale (NIHSS) change after 24 h [MD: 0.44 with 95% CI (0.0, 0.87), *P* = 0.05]. However, the intensive BP target was significantly associated with a decreased risk of excellent neurological recovery (mRS ≤ 1) [RR: 0.87 with 95% CI (0.76, 0.99), *P* = 0.03], functional independence (mRS ≤ 2) [RR: 0.81 with 95% CI (0.73, 0.90), *P* = 0.0001] and independent ambulation (mRS ≤ 3) [RR: 0.85 with 95% CI (0.79, 0.92), *P* < 0.0001].

**Conclusions:**

An intensive BP target after EVT is associated with worse neurological recovery and significantly decreased rates of functional independence and independent ambulation compared to the standard BP target. Therefore, the intensive BP target should be avoided after EVT for acute ischemic stroke.

**Supplementary Information:**

The online version of this article (https://doi.org/10.1007/s00062-024-01391-6) contains supplementary material, which is available to authorized users.

## Introduction

Stroke is the second-leading cause of death and the third-leading cause of death and disability combined worldwide [1]. The most common type is acute ischemic stroke (AIS), which accounts for 62.4% of new stroke cases and more than half of stroke deaths [1–3]. Endovascular thrombectomy (EVT) has become the standard of care for AIS patients with large vessel occlusion because it induces better functional autonomy than medical care [4–6].

Optimal blood pressure (BP) management after EVT is crucial to obtain optimal clinical results with successful recanalization. This is particularly true in the first 24 h after EVT due to the fact that elevated mean SBP in the first 24 h following EVT is correlated with an increased risk of intracranial hemorrhagic (ICH) transformation (i.e., higher NIH Stroke Scale score), early functional decline, and all-cause mortality at three months [7, 8].

The current guidelines of The American Heart Association (AHA)/American Stroke Association (ASA) recommend targeting an SBP value of ≤ 180 mm Hg among patients who underwent EVT for 24–48 h post-EVT [9]. However, these guidelines were based on the results of prior thrombolysis trials, which may not be valid for EVT, and they did not indicate which value below 180 mm Hg is the most preferred for SBP. Data regarding the SBP cutoff value, leading to the best efficacy and safety outcomes after EVT is highly heterogeneous. The BEST multicenter prospective cohort study revealed that the mean SBP that led to good neurological outcomes within 90 days following EVT was 138 mm Hg, whereas those who had poor outcomes displayed a mean SBP of 155 mm Hg [10]. Similarly, Matusevicius et al. found that SBP < 140 mm Hg was linked to lower odds for symptomatic ICH after unsuccessful recanalization compared to SBP ≥ 160 mm Hg [11]. By contrast, the recently published BEST-II Randomized Clinical Trial demonstrated that following endovascular intervention for AIS, SBP less than either 140 mm Hg or 160 mm Hg did not differ from the recommended target (≤ 180 mm Hg) in terms of utility score; however, the study suggested a low probability of benefit in lowering SBP targets [12].

Resolution of the controversy regarding the optimal BP target after EVT is crucial to provide guidance to clinicians to obtain optimal outcomes with successful recanalization after EVT. Therefore, in the present systematic review and meta-analysis, we reviewed the existing body of evidence to compare the efficacy and safety outcomes of intensive BP control (SBP < 140 mm Hg as target) versus the standard BP control (SBP < 140 mm Hg as target) after EVT for AIS.

## Methodology

### Protocol Registration

Our systematic review and meta-analysis strictly adhered to the guidelines outlined in the Preferred Reporting Items for Systematic Reviews and Meta-Analyses (PRISMA) statement [13] and followed the methodology detailed in the Cochrane Handbook for Systematic Reviews and Meta-Analyses [14]. The review protocol was registered and is publicly available on PROSPERO under the following ID: CRD42023463206.

### Data Sources & Search Strategy

Two reviewers (B.A. and M. A.) conducted an extensive electronic literature search by utilizing multiple databases, encompassing PubMed (MEDLINE), Web of Science, SCOPUS, the Cochrane Central Register of Controlled Trials (CENTRAL), medRXiv, and EMBASE from the inception to September 7th, 2023, through the following search strategy: “(″mechanical thrombectomy″ OR ″endovascular*″) AND (stroke OR ″cerebrovascular accident*″ OR ″brain vascular accident*″ OR ″brain ischemia″ OR ″brain infarction″) AND (″intensive blood pressure″ OR ″blood pressure control″ OR ″blood pressure lowering″ OR ″blood pressure management″ OR ″blood pressure target″)” without using any search limits. Further search details are outlined (Table S1).

### Eligibility Criteria

Randomized controlled trials (RCTs) comparing intensive BP target (SBP < 140 mm Hg) versus standard BP control (SBP < 180 mm Hg) in patients with AIS undergoing EVT were included. Our primary outcomes were the National Institutes of Health Stroke Scale (NIHSS) [15] change after 24 h and excellent neurological recovery defined as modified Rankin Scale (mRS ≤ 1) after three months [16]. Secondary outcomes were functional independence (mRS ≤ 2), independent ambulation (mRS ≤ 3), poor neurological recovery (mRS 4–6), all-cause mortality, any serious adverse events, any ICH, symptomatic ICH, and recurrent stroke.

### Study Selection

Search results from all the databases were imported to Covidence.org, and duplicates were removed automatically. The remaining records were screened independently by four authors (A.M. A., A.M., I.G., and U.K.), and any conflict between them was resolved by mutual consensus. The screening was done OVER two steps: (i) title and abstract screening to determine the relevance of the study for this meta-analysis, and (ii) a comprehensive full-text screening adhering to the predefined eligibility criteria to determine final eligibility for both qualitative and quantitative analysis.

### Data Extraction

Four reviewers (AMA, AM, IG, and UK) employed a pilot-tested Excel extraction sheet to extract the following data from the included RCTs: summary of included studies (name of the first author, publication year, study design, blinding, country of the study, total number of participants, BP target, main inclusion criteria, primary outcome, and follow up duration); baseline information {number of patients in each group, age, SBP, diastolic blood pressure (DBP), baseline medication (IV thrombolysis, anticoagulants, antiplatelets, and antihypertensives), and comorbidities [atrial fibrillation (AF), hypertension (HTN), heart failure (HF), diabetes mellitus (DM), coronary artery disease (CAD), and previous stoke/transient ischemic attack (TIA)]}; and study outcomes [NIHSS score change after 24 h, excellent neurological recovery (mRS 0–1), functional independence (mRS 0–2), independent ambulation (mRS 0–3), EQ-5D-3L score, poor neurological recovery (mRS 4–6), all-cause mortality, any serious adverse events, any ICH, symptomatic ICH, and recurrent stroke]. Conflicts were resolved by discussion among reviewers to attain consensus scores.

### Risk of Bias and Certainty of Evidence

Four reviewers (A.B., A.M. A., I.G., and M.A.E.) assessed the quality of the studies included in the research independently using the Cochrane ROB2 tool [17]. The domains that were evaluated included the risk of bias resulting from the randomization process, the risk of bias due to deviation from the intended intervention, the risk of bias due to missing outcome data, the risk of bias in measuring the outcome, and the risk of bias in selecting the reported results. In the event of any disagreements, the reviewers discussed the issues to attain a consensus score.

To appraise the quality of evidence, two reviewers (M. A. and B.A.) utilized the Grading of Recommendations Assessment, Development, and Evaluation (GRADE) guidelines [18, 19]. We considered inconsistency, imprecision, indirectness, publication bias, and risk of bias. The evaluation was carried out for each outcome, and the decisions made were justified and documented. Any discrepancies were settled through discussion between the reviewers.

### Statistical Analysis

RevMan v5.3 software was used to carry out statistical analysis [20]. To pool the results of dichotomous outcomes, we used the risk ratio (RR) while, for continuous outcomes, we used the mean difference (MD), both with a 95% confidence interval (CI). To assess heterogeneity, we employed the Chi-square and I‑square tests, where the Chi-square test assesses the presence of heterogeneity, and the I‑square test assesses its degree. We interpreted the I‑square test as follows: not significant for 0–40%, moderate heterogeneity for 30–60%, and substantial heterogeneity for 50–90%, following the Cochrane Handbook (chapter nine) [14]. We considered an alpha level below 0.1 for the Chi-square test to denote significant heterogeneity.

## Results

### Search Results and Study Selection

**Fig. 1:**
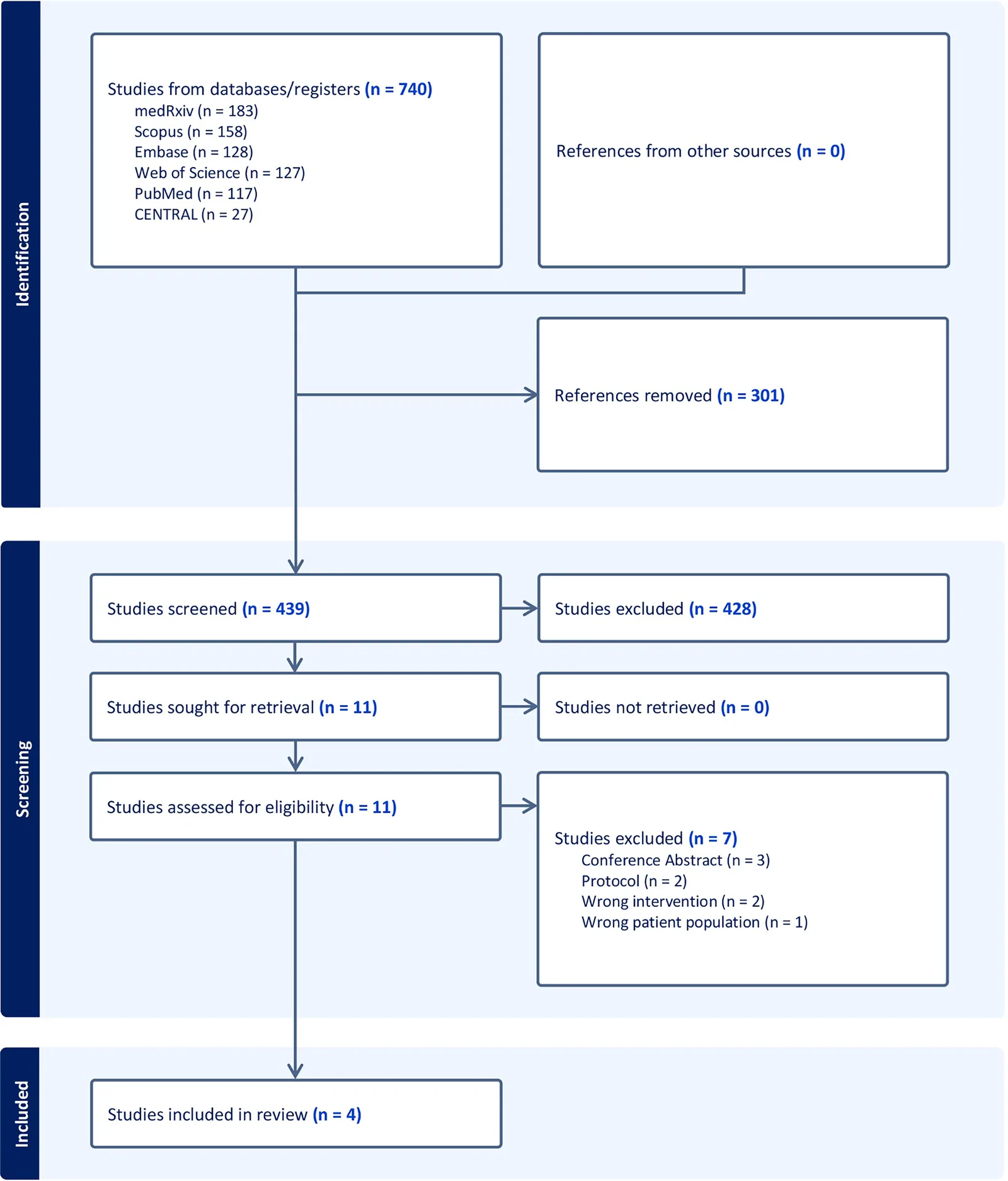
PRISMA flow chart of the screening process

We recaptured 740 records via searching five databases; 301 records of the 740 were duplicates and were automatically identified and removed via the Covidence online tool, leaving 439 records to screen. Of those; we eliminated 428 records in the title and abstract screening phase, leaving 11 studies to go through full-text screening. Finally, we identified studies that fulfilled our inclusion criteria and were incorporated into the qualitative and quantitative synthesis (Fig. 1).

### Characteristics of Included Studies

A total of four RCTs [12, 21–23] with 1559 participants have been included in the final analysis. All the four RCTs were open-label with three months of follow-up. In BP-Target, patients were randomized to BP targets within the first hour after recanalization, with the goal to reach and maintain BP target within one hour [21]; in BEST-II, patients were randomized to BP targets within 45 min after recanalization [12]; in The OPTIMAL BP, patients were randomized to BP targets within the two hours after recanalization [22]; and time from recanalization to randomization was not reported in ENCHANTED2/MT; however, the goal was to reach and maintain BP target within one hour after randomization [23]. All the included trials used nicardipine as the first-line antihypertensive agent to maintain the assigned target within one hour and for 24 h after randomization [12, 21, 22], except ENCHANTED2/MT, which mainly used urapidil [23]. Further details of the summary characteristics of the included RCTs and the baseline characteristics of the participants are outlined in (Tables 1 and 2, and S2–4).

**Table 1:** Summary characteristics of the included RCTs

Study ID	Study Design	Country	N. of Participants	SBP Target		Main Inclusion Criteria	Primary Outcome	Follow-up duration
				Intensive	Standard			
Mazighi et al. 2021 (BP-Target) [21]	Open-label, multicenter RCT	France	318	100–129 mm Hg	130–185 mm Hg	Adults with AIS due to anterior LVO (ICA, M1, or both) with successful reperfusion by EVT (defined as modified thrombolysis in cerebral infarction 2b or 3)	ICH at 24–36 h	Three months
Mistry et al. 2023 (BEST-II) [12]	Open-label, multicenter, blinded-endpoint, RCT	USA	120	< 140 mm Hg	< 160 mm Hg & ≤ 180 mm Hg	Adults with AIS due to anterior LVO (ICA, M1, or M2) with successful reperfusion by EVT (defined as modified thrombolysis in cerebral infarction 2b or greater)	Neurological disability assessment using mRS after three months	Three months
Nam et al. 2023 (The OPTIMAL BP) [22]	Open-label, multicenter, blinded-endpoint, RCT	South Korea	305	< 140 mm Hg	140–180 mm Hg	Adults with AIS due to LVO with successful reperfusion by EVT (defined as modified thrombolysis in cerebral infarction 2b or greater) and had elevated BP (SBP ≥ 140 mm Hg) according to at least two measurements within a two-minute interval within two hours of successful reperfusion	Neurological disability assessment using mRS after three months	Three months
Yang et al. 2022 (ENCHANTED2/MT) [23]	Open-label, multicenter, blinded-endpoint, RCT	China	816	< 120 mm Hg	140–180 mm Hg	Adults with AIS due to LVO with successful reperfusion by EVT (defined as modified thrombolysis in cerebral infarction 2b or greater) and had elevated BP (SBP ≥ 140 mm Hg) according to at least two measurements within three hours of successful reperfusion	Neurological disability assessment using mRS after three months	Three months

*RCT* randomized controlled trial, *SBP* systolic blood pressure, *ICH* intracranial hemorrhage, *AIS* acute ischemic stroke, *LVO* large vessel occlusion, *EVT* endovascular thrombectomy, *ICA* internal carotid artery, *M1* proximal middle cerebral artery, *M2* distal middle cerebral artery

**Table 2:** Baseline characteristics of the participants

Study ID	Number of patients in each group		Age (Years), Mean (SD)		Gender (Male), N. (%)		SBP, Mean (SD)		DBP, Mean (SD)		NIHSS, mean (SD)		Reperfusion (mTICI score), N. (%)					
	Intensive	Standard	Intensive	Standard	Intensive	Standard	Intensive	Standard	Intensive	Standard	Intensive	Standard	2B		2C		3	
													Intensive	Standard	Intensive	Standard	Intensive	Standard
Mazighi et al. 2021 (BP-Target) [21]	158	160	76 (14.22)	73.3 (14.96)	81 (51)	72 (45)	155 (26)	152 (25)	86 (18)	85 (15)	16.7 (5.99)	16.7 (5.24)	70 (44)	76 (48)	N/A	N/A	88 (56)	84 (52)
Mistry et al. 2023 (BEST-II) [12]	40	80	75.2 (13.84)	68.4 (12.57)	12 (30)	39 (48.75)	150 (22.5)	148.5 (24.16)	82.6 (14.5)	87.8 (18.93)	16.7 (9.23)	16.35 (5.89)	16 (40)	34 (42.5)	6 (15)	12 (15)	18 (45)	34 (42.5)
Nam et al. 2023 (The OPTIMAL BP) [22]	155	147	73.2 (12.1)	72.9 (10.8)	92 (59.4)	88 (59.9)	155.2 (13.4)	154.8 (14.4)	83.9 (14.4)	85.4 (14.1)	13 (6)	12 (7)	34 (21.9)	34 (22.7)	18 (11.6)	22 (14.7)	103 (66.5)	94 (62.7)
Yang et al. 2022 (ENCHANTED2/MT) [23]	407	409	68 (12)	67 (12)	249 (61)	257 (63)	158.1 (25)	158.7 (23)	89.4 (16)	89.5 (15)	15 (7.4)	15 (7.4)	37 (9)	43 (11)	28 (7)	26 (6)	342 (84)	340 (83)

*SBP* systolic blood pressure, *DBP* diastolic blood pressure, *NIHSS* National Institute of Health Stroke Scale, *N.* number, *SD* standard deviation, *N/A* not available, *mTICI* modified treatment in cerebral ischemia score

### Risk of Bias and Certainty of Evidence

**Fig. 2:**
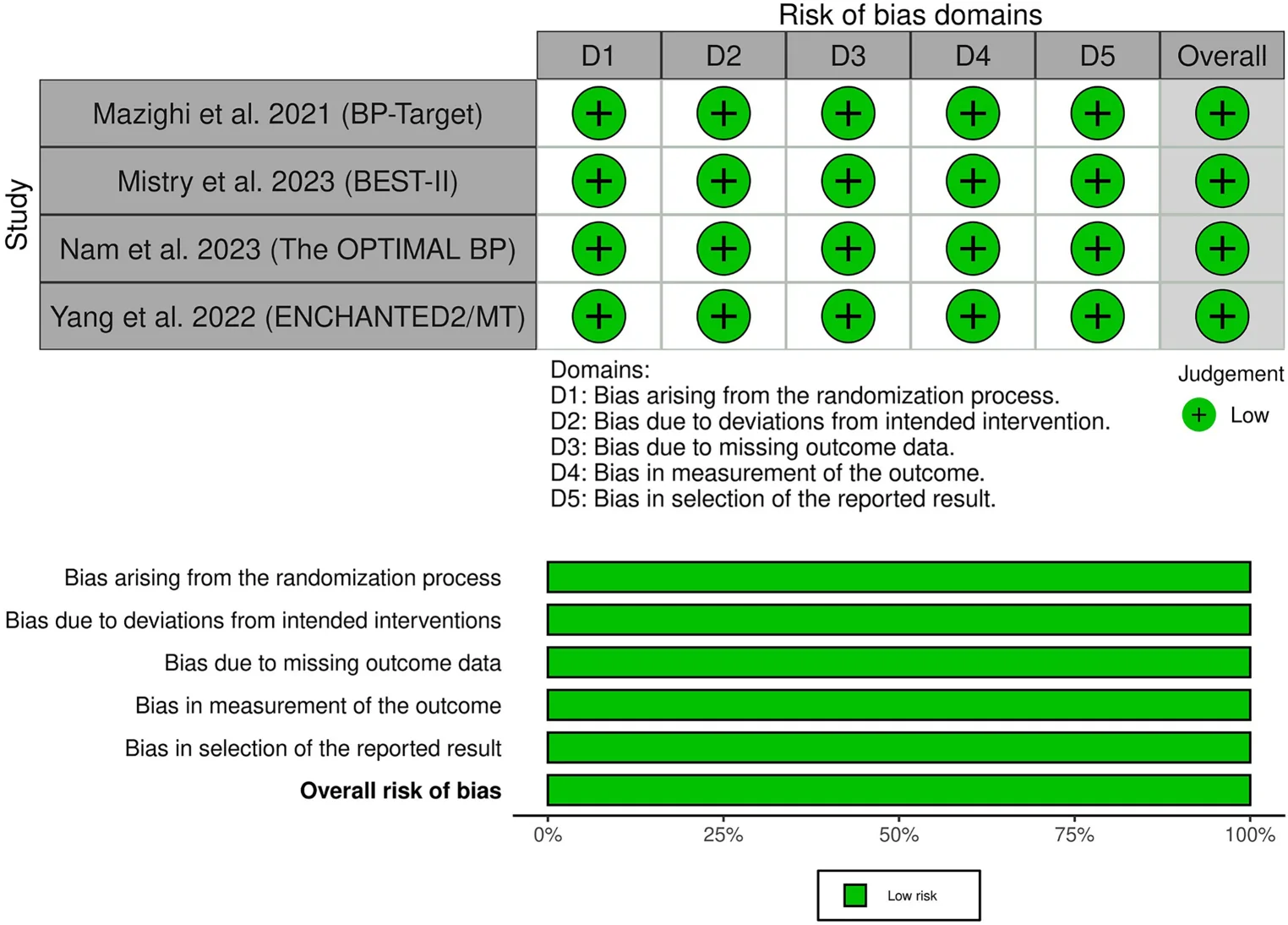
Quality assessment of risk of bias in the included trials. The upper panel presents a schematic representation of risks (low = green, unclear = yellow, and high = red) for specific types of biases of each of the studies in the review. The lower panel presents risks (low = green, unclear = yellow, and high = red) for the subtypes of biases of the combination of studies included in this review

All the included RCTs showed a low risk of bias, as detailed in (Fig. 2, Tables S5–8). Certainty of evidence is outlined in (Table 3).

**Table 3:** GRADE evidence profile of certainty of evidence

Certainty assessment							Summary of findings				
Participants (studies) Follow-up	Risk of bias	Inconsistency	Indirectness	Imprecision	Publication bias	Overall certainty of evidence	Study event rates (%)		Relative effect (95% CI)	Anticipated absolute effects	
							With Standard BP Target	With Intensive BP Target		Risk with Standard BP Target	Risk difference with Intensive BP Target
*NIHSS Score Change after 24 Hours*											
1557 (4 RCTs)	Not serious	Not serious	Not serious	Serious^a^	None	⨁⨁⨁◯ Moderate	799	758	–	The mean NIHSS Score Chnge after 24 Hours was *0*	MD *0.7 higher* (0.13 lower to 1.53 higher)
*Excellent Neurological Recovery (mRS 0–1)*											
1530 (4 RCTs)	Not serious	Not serious	Not serious	Serious^a^	None	⨁⨁⨁◯ Moderate	312/782 (39.9%)	263/748 (35.2%)	*RR 0.87* (0.75 to 1.01)	399 per 1000	*52 fewer per 1000* (from 100 fewer to 4 more)
*Functional Independence (mRS 0–2)*											
1530 (4 RCTs)	Not serious	Not serious	Not serious	Serious^a^	None	⨁⨁⨁◯ Moderate	432/782 (55.2%)	337/748 (45.1%)	*RR 0.82* (0.72 to 0.93)	552 per 1000	*99 fewer per 1000* (from 155 fewer to 39 fewer)
*Independent Ambulation (mRS 0–3)*											
1530 (4 RCTs)	Not serious	Not serious	Not serious	Not serious	None	⨁⨁⨁⨁ High	522/782 (66.8%)	427/748 (57.1%)	*RR 0.85* (0.79 to 0.92)	668 per 1000	*100 fewer per 1000* (from 140 fewer to 53 fewer)
*Poor Neurological Recovery (mRS 4–6)*											
1530 (4 RCTs)	Not serious	Not serious	Not serious	Serious^a^	None	⨁⨁⨁◯ Moderate	260/782 (33.2%)	321/748 (42.9%)	*RR 1.30* (1.14 to 1.48)	332 per 1000	*100 more per 1000* (from 47 more to 160 more)
*ALL-Cause Mortality*											
1541 (4 RCTs)	Not serious	Not serious	Not serious	Very serious^b^	None	⨁⨁◯◯ Low	102/788 (12.9%)	113/753 (15.0%)	*RR 1.15* (0.90 to 1.48)	129 per 1000	*19 more per 1000* (from 13 fewer to 62 more)
*Adverse Events—Any Serious Adverse Event*											
936 (2 RCTs)	Not serious	Not serious	Not serious	Very serious^b^	None	⨁⨁◯◯ Low	115/489 (23.5%)	118/447 (26.4%)	*RR 1.05* (0.85 to 1.31)	235 per 1000	*12 more per 1000* (from 35 fewer to 73 more)
*Adverse Events—Any Intracerebral Hemorrhage*											
1548 (4 RCTs)	Not serious	Not serious	Not serious	Not serious	None	⨁⨁⨁⨁ High	272/793 (34.3%)	274/755 (36.3%)	*RR 1.05* (0.92 to 1.20)	343 per 1000	*17 more per 1000* (from 27 fewer to 69 more)
*Adverse Events—Symptomatic Intracerebral Hemorrhage*											
1524 (4 RCTs)	Not serious	Not serious	Not serious	Very serious^b^	None	⨁⨁◯◯ Low	51/778 (6.6%)	56/746 (7.5%)	*RR 1.12* (0.78 to 1.62)	66 per 1000	*8 more per 1000* (from 14 fewer to 41 more)
*Adverse Events—Recurrent Stroke*											
1134 (2 RCTs)	Not serious	Not serious	Not serious	Extremely serious^b^	None	⨁◯◯◯ Very low	21/569 (3.7%)	25/565 (4.4%)	*RR 1.19* (0.68 to 2.08)	37 per 1000	*7 more per 1000* (from 12 fewer to 40 more)

*CI* confidence interval, *MD* mean difference, *RR* risk ratio

Explanations:

a. The confidence interval does not exclude the risk of appreciable harm or benefit

b. The confidence interval does not exclude the risk of appreciable harm or benefit and the number of events is < 300 event

### Efficacy Outcomes

**Fig. 3:**
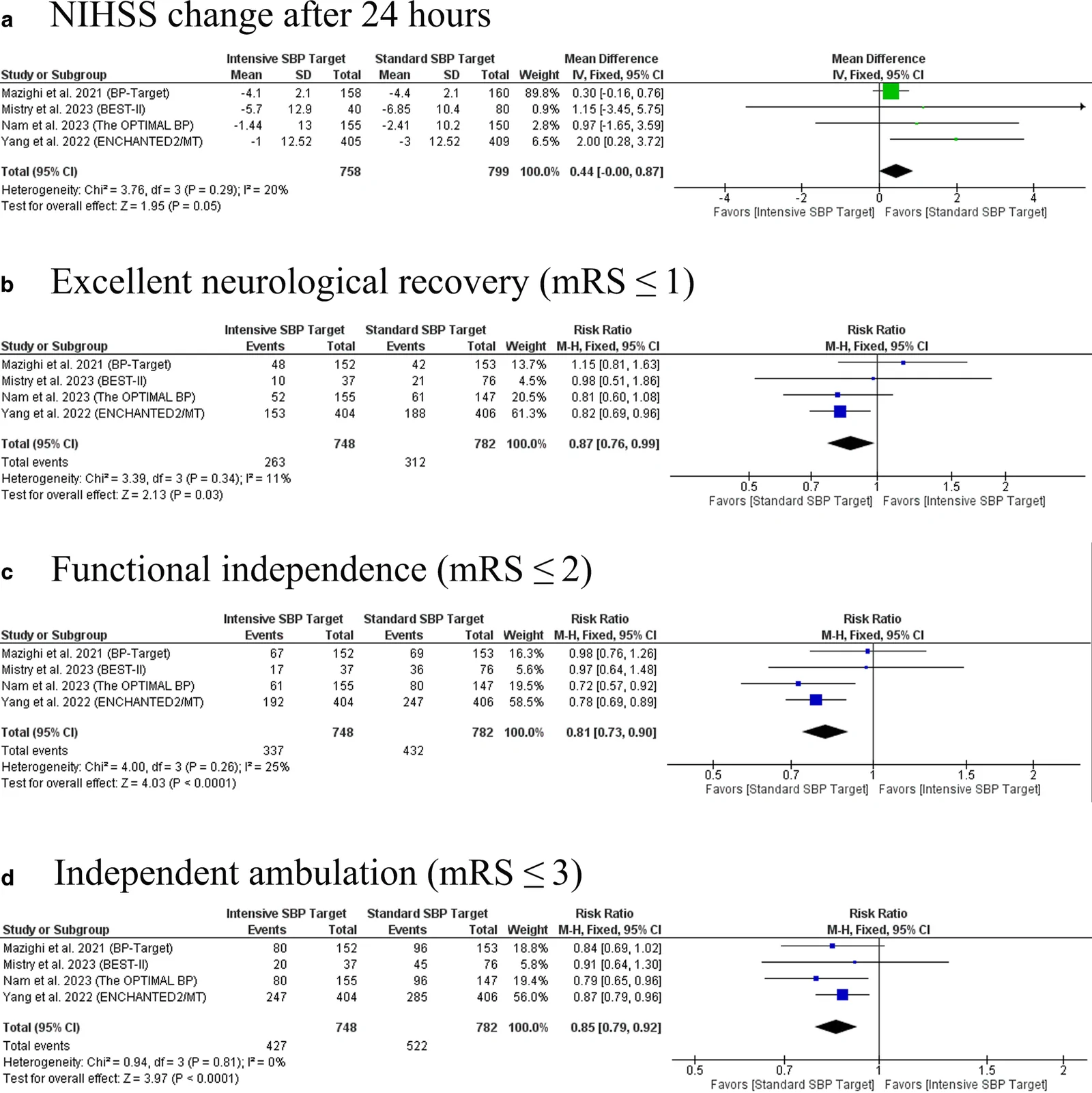
Forest plot of the efficacy outcomes, MD: mean difference, RR: risk ratio, CI: confidence interval

There was no difference between intensive BP and standard BP targets regarding NIHSS change after 24 h [MD: 0.44 with 95% CI (0.0, 0.87), *P* = 0.05] (Fig. 3a). However, the intensive BP target was significantly associated with a decreased risk of excellent neurological recovery (mRS ≤ 1) [RR: 0.87 with 95% CI (0.76, 0.99), *P* = 0.03] (Fig. 3b), functional independence (mRS ≤ 2) [RR: 0.81 with 95% CI (0.73, 0.90), *P* = 0.0001] (Fig. 3c) and independent ambulation (mRS ≤ 3) [RR: 0.85 with 95% CI (0.79, 0.92), P < 0.0001] (Fig. 3d).

Pooled studies were homogenous in NIHSS change (*P* = 0.29, I^2^ = 20%), excellent neurological recovery (mRS ≤ 1) (*P* = 0.34, I^2^ = 11%), functional independence (mRS ≤ 2) (*P* = 0.26, I^2^ = 25%), and independent ambulation (mRS ≤ 3) (*P* = 0.81, I^2^ = 0%).

### Safety Outcomes

**Fig. 4:**
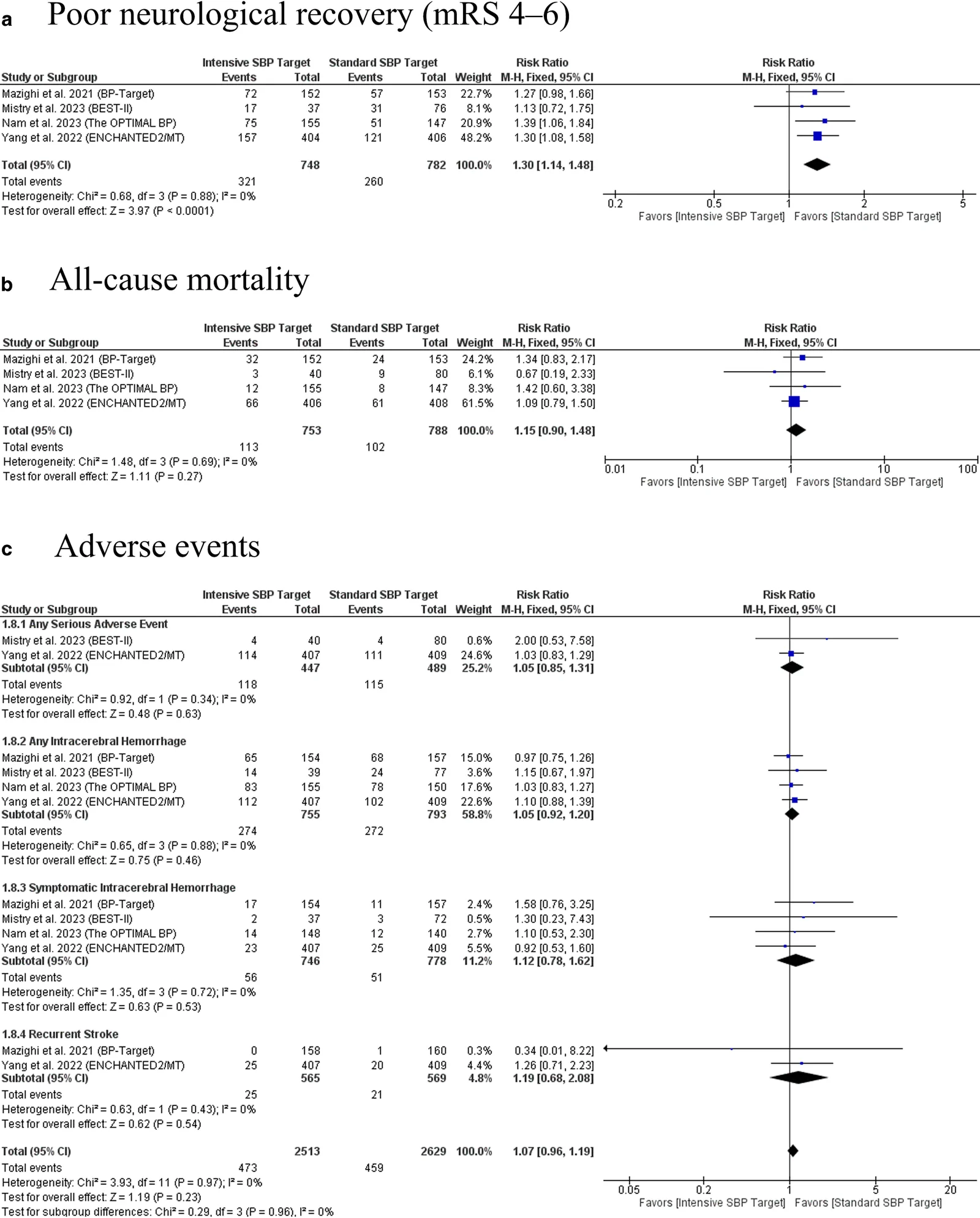
Forest plot of the safety outcomes, RR: risk ratio, CI: confidence interval

Intensive BP target was significantly associated with an increased rate of poor neurological recovery (mRS 4–6) [RR: 1.30 with 95% CI (1.14, 1.48), P < 0.0001] (Fig. 4a). Also, there was no difference between the two groups regarding the incidence of all-cause mortality [RR: 1.15 with 95% CI (0.90, 1.48), *P* = 0.27] (Fig. 4b), any serious adverse events [RR: 1.05 with 95% CI (0.85, 1.31), *P* = 0.63], any ICH [RR: 1.05 with 95% CI (0.92, 1.20), *P* = 0.46], symptomatic ICH [RR: 1.12 with 95% CI (0.78, 1.62), *P* = 0.53], and recurrent stroke [RR: 1.19 with 95% CI (0.68, 2.08), *P* = 0.63] (Fig. 4c).

Pooled studies were homogenous in poor neurological recovery (mRS 4–6) (*P* = 0.88, I^2^ = 0%), all-cause mortality (*P* = 0.69, I2 = 0%), any serious adverse events (*P* = 0.34, I^2^ = 0%), any ICH (*P* = 0.88, I^2^ = 0%), symptomatic ICH (*P* = 0.72, I^2^ = 0%), and recurrent stroke (*P* = 0.43, I^2^ = 0%).

## Discussion

In this systematic review and meta-analysis, we found with a moderate level of certainty that intensive BP control increases the risk of poor neurological outcomes after EVT for AIS. This manifested as significantly lower rates of excellent neurological recovery (mRS ≤ 1), functional independence (mRS ≤ 2), and independent ambulation (mRS ≤ 3), along with a higher risk of severe or fatal neurological disability (mRS 4–6) in the group with intensive BP target of < 140 mm Hg compared to that with the standard BP target of < 180 mm Hg. This supports the maintenance of current guidelines regarding the primary goals of BP after EVT if further lowering in the cutoff values did not provide any benefits; rather, it led to increased potential harm.

The examined evidence in this meta-analysis indicates that a substantial decrease in BP leads to more detrimental outcomes than a more modest control of the elevated BP following EVT. This is consistent with the previous results showing a positive correlation between considerable drops in BP among AIS patients undergoing EVT and worse neurological outcomes [24–27]. Two main mechanisms seem to be collectively or separately involved: (i) the loss of compensatory anti-ischemic effects of poststroke BP peak; and (ii) the increase in the vulnerability to cerebral hypoperfusion.

Thus, AIS is mostly due to a thrombotic or embolic obstruction of a single brain artery, resulting in a focal necrotic zone of cerebral infarction in which there is relatively no blood supply. This zone is surrounded by a hypoperfused, but still viable area called the “ischemic penumbra” in which low perfusion pressure leads to the creation of a pressure gradient force driving the retrograde movement of blood into the ischemic penumbral zone [28]. Hence, the ischemic penumbra is the target for cerebral reperfusion interventions as it represents a salvageable neuronal tissue with possible restoration of baseline function [29]. Notably, intensive control of poststroke BP was found to expand the cerebral infarction zone, likely by impeding the rise in the cerebral perfusion pressure [30], which is a key autoregulatory process necessary for maintaining adequate cerebral perfusion to the ischemic penumbra.

Furthermore, during AIS there is a possibility of cerebral autoregulation loss due to direct damage to the actin within the vascular wall or brainstem lesions (i.e., during vertebrobasilar stroke), producing local vasculo-paralysis, which ultimately exposes to a greater risk of hypoperfusion and low BP [31, 32]. Moreover, most AIS patients are elderly, which puts them at risk of aging-related impairment in the CBF regulation capacities [33]. Most AIS patients also have chronic hypertension, leading to a rightward shift in the autoregulation curve. This is a phenomenon that protects the cerebral microcirculation from the deleterious effects of chronic hypertension and possible volume overload through maintaining a constant CBF regardless of BP changes (i.e., by impairing endothelium-dependent relaxation, thereby, preventing vasodilatation of brain vasculature). However, this exposes to cerebral hypoperfusion even with modest control of BP [34]. Thereby, most AIS patients can be at a vulnerable condition to hypoperfusion during brain ischemic events, and an intensive BP lowering would increase this vulnerability.

### Implications for Future Practice & Research

Careful management of BP after EVT is crucial to reduce the risk of undesired neurological outcomes. Given the fact that current guidelines recommend a drop in the SBP of < 180 mm Hg for 24 to 48 h post-EVT [9] and that this drop should not lead to values < 140 mm Hg as shown in our study, it makes the BP control in the context of EVT a real multifaceted challenge. Therefore, future research should investigate effective strategies to be used to help accomplish the targeted BP control in EVT patients. Thus, this can mainly be achieved by strict monitoring of BP and adequate anti-hypertensive therapy (not too aggressive, not too permissive). BP fluctuations are a common phenomenon in the poststroke acute phase likely due to autonomic dysregulation, and are associated with unfavorable outcomes [35, 36]. Consequently, this should be considered to optimize the quality of monitoring among the frequently hemodynamically unstable AIS patients.

Regarding the anti-hypertensive agent of choice, previous reports suggested short and rapidly acting intravenous drugs to be preferred, parsticularly labetalol, hydralazine, esmolol, nicardipine, enalapril, nitroglycerin, and nitroprusside, which have been recommended in AIS patients in the United States. Additionally, intravenous urapidil is also used in Europe [37]. Nonetheless, data regarding the protocol of anti-hypertensive therapy (agent, dose, modality) that would provide the best BP control in the acute phase following EVT is lacking and requires investigations.

Another promising approach that should be investigated is personalized BP control, which may offer more patient-centered management. Thus, continuous measuring of the autoregulatory function of stroke patients who have undergone EVT was previously achieved by recording modifications of the near-infrared spectroscopy-derived tissue oxygenation (a CBF surrogate) in response to changes in mean BP [38]. Interestingly, this enabled the non-invasive determination of personalized BP thresholds, which when exceeded, had led to an increased risk of further brain injury and poor functional outcome. Recently, individualized BP management among EVT patients was found to induce similar rates of favorable functional status at three months compared with the standard BP management [39].

Finally, for AIS patients treated with IV thrombolysis, the pioneer ENCHANTED trial revealed that intensive BP control displays no superiority over the standard BP lowering regarding improving clinical recovery, despite the reduction in ICH risk [40]. Moreover, Wang et al. did not identify any beneficial interaction between low-dose alteplase and intensive BP-lowering therapy in reducing ICH [41]. However, a post hoc secondary analysis of the ENCHANTED trial’s data suggested a ≈20% reduction in the odds of unfavorable functional outcomes for every 10 mm Hg decrease in the SBP below 110 to 120 mm Hg early after symptom onset [42]. Therefore, further studies are still required to provide conclusions on this issue.

### Strengths & Limitations

To the best of our knowledge, this meta-analysis is the first to evaluate the efficacy and safety of intensive BP control versus the standard BP control among AIS patients who underwent EVT. After an extensive search, we covered data from all published RCTs which included records of 1599 patients. The examined pooled studies were homogenous, and the findings were insightful and of important clinical implications and acceptable certainty of evidence. Nevertheless, our work was prone to some limitations. First, the intensive SBP target varied among the included trials from < 120 to < 140 mm Hg, which can affect our findings, and subgroup analysis based on the SBP target was not feasible due to the paucity of the available data. Second, a lack of long-term data as the follow-up of the included trials did not exceed three months. Third, the open-label design of all the included RCTs would considerably underpowered their findings despite the low risk of performance bias. Fourth, the generalizability concerns due to the recruited clinical trial population being exclusively from developed countries (USA, China, France, South Korea) where cerebral atherosclerosis and hypertension are highly prevalent. Since patients were either from Western or Eastern countries, there is a potential for ethnic differences that would also compromise the generalizability of the trial results. Fifth, this is an aggregate-based, not an individual patient data-based meta-analysis, which would have enabled us to assess some confounding variables such as reperfusion rates and time in BP target. Finally, the issue of not addressing the SBP management before the EVT.

## Conclusion

Intensive BP control after EVT for AIS led to an increased risk of unfavorable post-interventional outcomes. Therefore, intensive BP lowering should be avoided after EVT. Instead, management of BP should aim to maintain SBP values less than 180 mm Hg and higher than 140 mm Hg, which seems so far the optimal BP target after EVT. However, since achieving this interval in the acute setting of AIS is challenging, future studies should investigate how to improve BP control and monitoring after EVT.

## Supplementary Information

Supplementary tables, mainly outlining the search strategy and the quality assessment details.

## References

[1] Feigin VL, Stark BA, Johnson CO, Roth GA, Bisignano C, Abady GG, et al. Global, regional, and national burden of stroke and its risk factors, 1990–2019: A systematic analysis for the Global Burden of Disease Study 2019. Lancet Neurol England. 2021;20:1–26. 10.1016/S1474-4422(21)00252-0 PMC8443449 34487721

[2] Fan J, Li X, Yu X, Liu Z, Jiang Y, Fang Y, et al. Global Burden, Risk Factor Analysis, and Prediction Study of Ischemic Stroke, 1990–2030. Neurology. Wolters Kluwer Health, Inc. on behalf of the American Academy of Neurology. 2023;101:e137–e150. 10.1212/WNL.0000000000207387 PMC10351546 37197995

[3] Abuelazm M, Seri AR, Awad AK, Ahmad U, Mahmoud A, Albazee E, et al. The efficacy and safety of tenecteplase versus alteplase for acute ischemic stroke: an updated systematic review, pairwise, and network meta-analysis of randomized controlled trials. J Thromb Thrombolysis. 2023;55:322–38. 10.1007/s11239-022-02730-5 PMC10011306 36449231

[4] Abuelazm M, Ahmad U, Suilik AH, Seri A, Mahmoud A, Abdelazeem B. Endovascular Thrombectomy for Acute Stroke with a Large Ischemic Core: A Systematic Review and Meta-Analysis of Randomized Controlled Trials. Clin Neuroradiol. 2023;. 10.1007/s00062-023-01306-x PMC10450014 37233795

[5] Sarraj A, Kleinig TJ, Hassan AE, Portela PC, Ortega-Gutierrez S, Abraham MG, et al. Association of Endovascular Thrombectomy vs Medical Management With Functional and Safety Outcomes in Patients Treated Beyond 24 Hours of Last Known Well: The Select Late Study. JAMA Neurol. United States; 2023;80:172–82. 10.1001/jamaneurol.2022.4714 PMC9857518 36574257

[6] Morsi RZ, Elfil M, Ghaith HS, Aladawi M, Elmashad A, Kothari S, et al. Endovascular Thrombectomy for Large Ischemic Strokes: A Living Systematic Review and Meta-Analysis of Randomized Trials. J Stroke. 2023;25:214–22. 10.5853/jos.2023.00752 PMC10250873 37282371

[7] Katsanos AH, Malhotra K, Ahmed N, Seitidis G, Mistry EA, Mavridis D, et al. After Endovascular Thrombectomy and Outcomes in Patients With Acute Ischemic Stroke: An Individual Patient Data Meta-analysis. Neurology. United States. Blood Press. 2022;98:e291–301. 10.1212/WNL.0000000000013049 34772799

[8] Matusevicius M, Cooray C, Bottai M, Mazya M, Tsivgoulis G, Nunes AP, et al. Blood Pressure After Endovascular Thrombectomy. Stroke. 2020;51:519–25. 10.1161/STROKEAHA.119.026914 31822252

[9] Powers WJ, Rabinstein AA, Ackerson T, Adeoye OM, Bambakidis NC, Becker K, et al. Guidelines for the Early Management of Patients With Acute Ischemic Stroke: 2019 Update to the 2018 Guidelines for the Early Management of Acute Ischemic Stroke: A Guideline for Healthcare Professionals From the American Heart Association/American Stroke. Stroke. United States; 2019. p. e344–418. 10.1161/STR.0000000000000211 31662037

[10] Mistry EA, Mehta T, Mistry A, Arora N, Starosciak AK, De La Los Rios RF, et al. Variability and Neurologic Outcome After Endovascular Thrombectomy: A Secondary Analysis of the BEST Study. Stroke. United States. Blood Press. 2020;51:511–8. 10.1161/STROKEAHA.119.027549 PMC8010595 31813361

[11] Matusevicius M, Cooray C, Bottai M, Mazya M, Tsivgoulis G, Nunes AP, et al. After Endovascular Thrombectomy: Modeling for Outcomes Based on Recanalization Status. Stroke. United States. Blood Press. 2020;51:519–25. 10.1161/STROKEAHA.119.026914 31822252

[12] Mistry EA, Hart KW, Davis LT, Gao Y, Prestigiacomo CJ, Mittal S, et al. Blood Pressure Management After Endovascular Therapy for Acute Ischemic Stroke: The BEST-II Randomized Clinical Trial. Jama [Internet]. 2023;330:821–31. Available from: http://www.ncbi.nlm.nih.gov/pubmed/37668620. 10.1001/jama.2023.14330 PMC10481231 37668620

[13] Page MJ, McKenzie JE, Bossuyt PM, Boutron I, Hoffmann TC, Mulrow CD, et al. The PRISMA 2020 statement: an updated guideline for reporting systematic reviews. BMJ [Internet]. British Medical Journal Publishing Group; 2021 [cited 2021 Aug 13];372. Available from: https://www.bmj.com/content/372/bmj.n71. 10.1136/bmj.n71 PMC8005924 33782057

[14] Higgins JPT, Thomas J, Chandler J, Cumpston M, Li T, Page MJ, et al. Cochrane handbook for systematic reviews of interventions. Cochrane Handb Syst Rev Interv. 2019. pp. 1–694. 10.1002/14651858.ED000142 PMC10284251 31643080

[15] Chalos V, van der Ende NAM, Lingsma HF, Mulder MJHL, Venema E, Dijkland SA, et al. National Institutes of Health Stroke Scale: An Alternative Primary Outcome Measure for Trials of Acute Treatment for Ischemic Stroke. Stroke [Internet]. Wolters Kluwer Health; 2020 [cited 2023 Sep 25];51:282. 10.1161/STROKEAHA.119.026791. PMC6924951 31795895

[16] JL B, CA M. Outcomes Validity and Reliability of the Modified Rankin Scale: Implications for Stroke Clinical Trials A Literature Review and Synthesis. Stroke. 2007;38:1091–6. 10.1161/01.STR.0000258355.23810.c6 17272767

[17] Sterne JAC, Savović J, Page MJ, Elbers RG, Blencowe NS, Boutron I, et al. RoB 2: a revised tool for assessing risk of bias in randomised trials. BMJ [Internet]. British Medical Journal Publishing Group; 2019 [cited 2021 Jul 22];366. Available from: https://www.bmj.com/content/366/bmj.l4898. 10.1136/bmj.l4898 31462531

[18] Guyatt GH, Oxman AD, Kunz R, Vist GE, Falck-Ytter Y, Schünemann HJ. Rating Quality of Evidence and Strength of Recommendations: What is “quality of evidence” and why is it important to clinicians? BMJ Br Med J [Internet]. BMJ Publishing Group; 2008 [cited 2021 Dec 23];336:995. 10.1136/bmj.39490.551019.BE.

[19] Guyatt GH, Oxman AD, Vist GE, Kunz R, Falck-Ytter Y, Alonso-Coello P, et al. Rating Quality of Evidence and Strength of Recommendations: GRADE: an emerging consensus on rating quality of evidence and strength of recommendations. BMJ Br Med J [Internet]. BMJ Publishing Group; 2008 [cited 2021 Dec 23];336:924. 10.1136/bmj.39489.470347.AD. PMC2335261 18436948

[20] RevMan | Cochrane Training [Internet]. [cited 2021 Aug 3]. Available from: https://training.cochrane.org/online-learning/core-software-cochrane-reviews/revman.

[21] Mazighi M, Richard S, Lapergue B, Sibon I, Gory B, Berge J, et al. Safety and efficacy of intensive blood pressure lowering after successful endovascular therapy in acute ischaemic stroke (BP-TARGET): a multicentre, open-label, randomised controlled trial. Lancet Neurol. 2021;20:265–74. 10.1016/S1474-4422(20)30483-X 33647246

[22] Nam HS, Kim YD, Heo J, Lee H, Jung JW, Choi JK, et al. Intensive vs Conventional Blood Pressure Lowering After Endovascular Thrombectomy in Acute Ischemic Stroke: The OPTIMAL-BP Randomized Clinical Trial. Jama [Internet]. 2023;330:832–42. Available from: http://www.ncbi.nlm.nih.gov/pubmed/37668619. 10.1001/jama.2023.14590 PMC10481233 37668619

[23] Yang P, Song L, Zhang Y, Zhang X, Chen X, Li Y, et al. Intensive blood pressure control after endovascular thrombectomy for acute ischaemic stroke (ENCHANTED2/MT): a multicentre, open-label, blinded-endpoint, randomised controlled trial. Lancet. 2022;400:1585–96. 10.1016/S0140-6736(22)01882-7 36341753

[24] Petersen NH, Ortega-Gutierrez S, Wang A, Lopez G V, Strander S, Kodali S, et al. Decreases in Blood Pressure During Thrombectomy Are Associated With Larger Infarct Volumes and Worse Functional Outcome. Stroke. United States; 2019;50:1797–804. 10.1161/STROKEAHA.118.024286 PMC6787912 31159701

[25] Maïer B, Dargazanli C, Bourcier R, Kyheng M, Labreuche J, Mosimann PJ, et al. Effect of Steady and Dynamic Blood Pressure Parameters During Thrombectomy According to the Collateral Status. Stroke. United States; 2020;51:1199–206. 10.1161/STROKEAHA.119.026769 32156204

[26] Maïer B, Robichon E, Bourcier R, Dargazanli C, Labreuche J, Thion L‑A, et al. Association of Hypotension During Thrombectomy and Outcomes Differs With the Posterior Communicating Artery Patency. Stroke United states;. 2021;52:2964–7. 10.1161/STROKEAHA.121.034542 34134507

[27] Löwhagen Hendén P, Rentzos A, Karlsson J‑E, Rosengren L, Sundeman H, Reinsfelt B, et al. Hypotension During Endovascular Treatment of Ischemic Stroke Is a Risk Factor for Poor Neurological Outcome. Stroke United states;. 2015;46:2678–80. 10.1161/STROKEAHA.115.009808 26173727

[28] Peng TJ, Ortega-Gutiérrez S, de Havenon A, Petersen NH. Management After Endovascular Thrombectomy. Front Neurol. Switzerland. Blood Press. 2021;12:723461. 10.3389/fneur.2021.723461 PMC8446280 34539562

[29] Regenhardt RW, Das AS, Stapleton CJ, Chandra RV, Rabinov JD, Patel AB, et al. Blood Pressure and Penumbral Sustenance in Stroke from Large Vessel Occlusion. Front Neurol Switzerland;. 2017;8:317. 10.3389/fneur.2017.00317 PMC5494536 28717354

[30] Thakkar P, McGregor A, Barber PA, Paton JFR, Barrett C, McBryde F. Hypertensive Response to Ischemic Stroke in the Normotensive Wistar Rat. Stroke United states;. 2019;50:2522–30. 10.1161/STROKEAHA.119.026459 31449479

[31] Cipolla MJ, Lessov N, Hammer ES, Curry AB. Threshold duration of ischemia for myogenic tone in middle cerebral arteries: effect on vascular smooth muscle actin. Stroke. United States; 2001;32:1658–64. 10.1161/01.str.32.7.1658 11441216

[32] Tamayo A, Siepmann T. Regulation of Blood Flow in the Cerebral Posterior Circulation by Parasympathetic Nerve Fibers: Physiological Background and Possible Clinical Implications in Patients With Vertebrobasilar Stroke. Front Neurol Switzerland;. 2021;12:660373. 10.3389/fneur.2021.660373 PMC8585859 34777191

[33] Tarumi T, Zhang R. Cerebral blood flow in normal aging adults: cardiovascular determinants, clinical implications, and aerobic fitness. J Neurochem England;. 2018;144:595–608. 10.1111/jnc.14234 PMC5874160 28986925

[34] Ruland S, Aiyagari V. Cerebral autoregulation and blood pressure lowering. Hypertens. Dallas, Tex., Vol. 1979. United States. 2007. pp. 977–8. 10.1161/HYPERTENSIONAHA.107.087502 17353508

[35] Yang C, Liu K, Song Y, Gong S, Ye R, Zhang Z, et al. Day-by-Day Blood Pressure Variability Is Associated With Neurological Functional Outcome After Acute Ischemic Stroke. Front Neurol. Switzerland; 2020;11:566825. 10.3389/fneur.2020.566825 PMC7691487 33281703

[36] Yong M, Kaste M. Association of characteristics of blood pressure profiles and stroke outcomes in the ECASS-II trial. Stroke. United States; 2008;39:366–72. 10.1161/STROKEAHA.107.492330 18096843

[37] Aiyagari V, Gorelick PB. Management of Blood Pressure for Acute and Recurrent Stroke. Stroke. 2009;40:2251–6. 10.1161/STROKEAHA.108.531574 19390077

[38] Wilson CP, Chakraborty AR, Pelargos PE, Shi HH, Milton CK, Sung S, et al. Rosette-forming glioneuronal tumor: an illustrative case and a. Syst Rev Neuro-oncology Adv. 2020;2. 10.1093/noajnl/vdaa116 PMC7586144 33134925

[39] Chen M, Meis J, Potreck A, Sauer LD, Kieser M, Bendszus M, et al. Effect of Individualized Versus Standardized Blood Pressure Management During Endovascular Stroke Treatment on Clinical Outcome: A Randomized Clinical Trial. Stroke. 2023;54:2755–65. 10.1161/STROKEAHA.123.044062 37732489

[40] Anderson CS, Huang Y, Lindley RI, Chen X, Arima H, Chen G, et al. Intensive blood pressure reduction with intravenous thrombolysis therapy for acute ischaemic stroke (ENCHANTED): an international, randomised, open-label, blinded-endpoint, phase 3 trial. Lancet. 2019;393:877–88. 10.1016/S0140-6736(19)30038-8 30739745

[41] Wang X, Song L, Yang J, Sun L, Moullaali TJ, Sandset EC, et al. Interaction of Blood Pressure Lowering and Alteplase Dose in Acute Ischemic Stroke: Results of the Enhanced Control of Hypertension and Thrombolysis Stroke Study. Cerebrovasc Dis. 2020;48:207–16. 10.1159/000504745 31812956

[42] Wang X, Minhas JS, Moullaali TJ, Di Tanna LG, Lindley RI, Chen X, et al. Associations of Early Systolic Blood Pressure Control and Outcome after Thrombolysis-Eligible Acute Ischemic Stroke: Results from the ENCHANTED Study. Stroke. 2022;29:779–87. 10.1161/STROKEAHA.121.034580 34702064

