# Supplementary material for: Intensive Blood Pressure Control After Endovascular Thrombectomy for Acute Ischemic Stroke: a Systematic Review and Meta-Analysis
Source: Clin Neuroradiol. 2024 Mar 7;34(3):563–75. doi: 10.1007/s00062-024-01391-6 (PMC11339153; doi:10.1007/s00062-024-01391-6)
Supplement: Supplementary file 1 — Supplementary tables, mainly outlining the search strategy and the quality assessment details. [file 62_2024_1391_MOESM1_ESM.docx]

**Supplementary material:**

**Title.**

**Intensive Blood Pressure Control after Endovascular Thrombectomy for Acute Ischemic Stroke: A Systematic Review and Meta-Analysis.**

**Running Title.**

**Intensive Blood Pressure** **Control after EVT.**

**Authors.**

Mohamed Abuelazm^a,*^, Yehya Khildj^b,*^, Ahmed A. Ibrahim^c^, Abdelrahman Mahmoud^d^, Ahmed Mazen Amin^e^, Ibrahim Gowaily^a^, Ubaid Khan^f^, Basel Abdelazeem^g^, James Robert Brašić^h-j^

**Affiliations.**

^a^ Faculty of Medicine, Tanta University, Tanta, Egypt

^b^Faculty of Medicine, University of Algiers, Algiers, Algeria

^c^Facultyof Medicine, Menoufia University, Menoufia, Egypt

^d^Faculty of Medicine, Minia University, Minia, Egypt

^e^Faculty of Medicine, Mansoura University, Mansoura, Egypt

^f^Faculty of Medicine, King Edward Medical University, Lahore, Pakistan

^g^Department of Cardiology, West Virginia University, West Virginia, USA

^h^Section of High-Resolution Brain Positron Emission Tomography Imaging, Division of Nuclear Medicine and Molecular Imaging, The Russell H. Morgan Department of Radiology and Radiological Science, The Johns Hopkins University School of Medicine, Baltimore, MD, USA

^i^Department of Behavioral Health, New York City Health and Hospitals/Bellevue, New York, NY, USA

^j^Department of Psychiatry, New York University Grossman School of Medicine, New York University Langone Health, New York, NY, USA

*, Both authors have equal contributions and are co-first authors.

**Contents:**

**Tables.**Table S1: Search strategy.

Table S2: Medical and medication history of the included participants.

Table S3: Blood pressure montoring details of the included trials.

Table S4: Type of the used anti-hypertensives.

Table S5: Risk of bias assessment details of Mazighi et al. 2021 (BP-Target), using ROB2.

Table S6: Risk of bias assessment details of Mistry et al. 2023 (BEST-II), using ROB2.

Table S7: Risk of bias assessment details of Nam et al. 2023 (The OPTIMAL BP), using ROB2.

Table S8: Risk of bias assessment details of Yang et al. 2022 (ENCHANTED2/MT), using ROB2.

| Database | Search Terms | Search Field | Search Results |
| --- | --- | --- | --- |
| Pubmed | ("mechanical thrombectomy" OR "endovascular*") AND (stroke OR "cerebrovascular accident*" OR "brain vascular accident*" OR ”brain ischemia” OR”'brain infarction”) AND (”intensive blood pressure” OR ”blood pressure control” OR ”blood pressure lowering” OR ”blood pressure management” OR ”blood pressure target”) | All Fields | 117 |
| Cochrane | ("mechanical thrombectomy" OR "endovascular thrombectomy") AND (stroke OR "cerebrovascular accident" OR "brain vascular accident" OR "brain ischemia" OR "brain infarction") AND (”intensive blood pressure” OR ”blood pressure control” OR ”blood pressure lowering” OR ”blood pressure management” OR ”blood pressure target”) | All Fields | 27 |
| WOS | ("mechanical thrombectomy" OR "endovascular*") AND (stroke OR "cerebrovascular accident*" OR "brain vascular accident*" OR ”brain ischemia” OR”'brain infarction”) AND (”intensive blood pressure” OR ”blood pressure control” OR ”blood pressure lowering” OR ”blood pressure management” OR ”blood pressure target”) | All Fields | 127 |
| Scopus | ("mechanical thrombectomy" OR "endovascular*") AND (stroke OR "cerebrovascular accident*" OR "brain vascular accident*" OR ”brain ischemia” OR”'brain infarction”) AND (”intensive blood pressure” OR ”blood pressure control” OR ”blood pressure lowering” OR ”blood pressure management” OR ”blood pressure target”) | Title, Abstract, Keywords | 158 |
| EMBASE | #4. #1 AND #2 AND #3  #3. 'intensive blood pressure':ti,ab,kw OR 'blood  pressure regulation':ti,ab,kw OR 'blood pressure  lowering':ti,ab,kw OR 'blood pressure  management':ti,ab,kw OR 'blood pressure  target':ti,ab,kw  #2. stroke:ti,ab,kw OR 'cerebrovascular  accident':ti,ab,kw OR 'brain vascular  accident*':ti,ab,kw OR 'brain ischemia':ti,ab,kw  OR 'brain infarction':ti,ab,kw  #1. 'mechanical thrombectomy':ti,ab,kw OR  endovascular:ti,ab,kw | All Fields | 128 |
| medRXiv | ("mechanical thrombectomy" OR "endovascular thrombectomy") AND (stroke OR "cerebrovascular accident" OR "brain vascular accident" OR "brain ischemia" OR "brain infarction") AND (”intensive blood pressure” OR ”blood pressure control” OR ”blood pressure lowering” OR ”blood pressure management” OR ”blood pressure target”) | All Fields | 183 |

Table S1: Search Strategy.

| Study ID | Past medical history, N. (%) | | | | | | | | | | Medications History N. (%) | | | | | | | |
| --- | --- | --- | --- | --- | --- | --- | --- | --- | --- | --- | --- | --- | --- | --- | --- | --- | --- | --- |
|  | Hyperlipidemia | | Hypertension | | Atial fibrillation | | Diabetes | | Current smoking | | Antiplatelet | | Anticoagulation drug | | Antihypertensive drugs | | Statin or other lipid-lowering drug | |
|  | **Intensive** | **Standard** | **Intensive** | **Standard** | **Intensive** | **Standard** | **Intensive** | **Standard** | **Intensive** | **Standard** | **Intensive** | **Standard** | **Intensive** | **Standard** | **Intensive** | **Standard** | **Intensive** | **Standard** |
| Mazighi et al. 2021 (BP-Target) | 59 (39) | 55(35) | 110 (70) | 113 (71) | NA | NA | 34 (22) | 33 (21) | 19 (14) | 24 (16) | 44 (28) | 37 (23) | 36 (23) | 34 (21) | 101 (66) | 102 (64) | NA | NA |
| Mistry et al. 2023 (BEST-II) | 33.0 (82.5) | 28 (70) | 32 (80) | 28 (70) | 19 (47.5) | 13 (32.5) | 12 (30) | 15 (37.5) | 8 (20) | 12 (30) | 14 (35) | 13 (32.5) | 10 (25) | 3 (7.5) | 27 (67.5) | 26 (65) | NA | NA |
| Nam et al. 2023 (The OPTIMAL BP) [ | 61 (39.4) | 54 (36.7) | 121 (78.1) | 110 (74.8) | 77 (49.7) | 69 (46.9) | 65 (41.9) | 62 (42.2) | 39 (25.2) | 29 (19.7) | NA | NA | NA | NA | NA | NA | NA | NA |
| Yang et al. 2022 (ENCHANTED2/MT) | 14 (3) | 13 (3) | 267 (66) | 261 (64) | 84 (21) | 98 (24) | 81 (20) | 82 (20) | NA | NA | 34 (8) | 39 (10) | 20 (5) | 20 (5) | 176 (43) | 179 (44) | 30 (7) | 30 (7) |

Table S2: Medical and medication history of the included participants.

N., number; N/A, not available.

| **Study ID** | **BP parameters,**  **mean (SD), mmHg** | | | | | | | | | | | | **Time in target SBP range,** **N. (%)** | | | | | | **SBP out of range, N. (%)** | | | | | |
| --- | --- | --- | --- | --- | --- | --- | --- | --- | --- | --- | --- | --- | --- | --- | --- | --- | --- | --- | --- | --- | --- | --- | --- | --- |
|  | SBP at 1h | | DBP at 1h | | SBP at 24 h | | DBP at 24 h | | Mean SBP over 24 h | | Mean DBP over 24 h | | SBP <140 mmHg) | | SBP 140-180 mmHg | | SBP <180 mmHg) | | SBP exceeding >180 mmHg at least once | | SBP exceeding >200 mmHg at least once | | SBP dropping <100 mmHg at least once | |
|  | Intensive | Standard | Intensive | Standard | Intensive | Standard | Intensive | Standard | Intensive | Standard | Intensive | Standard | Intensive | Standard | Intensive | Standard | Intensive | Standard | Intensive | Standard | Intensive | Standard | Intensive | Standard |
| Mazighi et al. 2021 (BP-Target) | NA | NA | NA | NA | NA | NA | NA | NA | 128 (11) | 138 (17) | NA | NA | NA | NA | NA | NA | NA | NA | NA | NA | NA | NA | 2(1) | 1(0.6) |
| Mistry et al. 2023 (BEST-II) | NA | NA | NA | NA | NA | NA | NA | NA | NA | NA | NA | NA | NA | NA | NA | NA | NA | NA | NA | NA | NA | NA | NA | NA |
| Nam et al. 2023 (The OPTIMAL BP) [ | 135.1 (19.9) | 140.6 (19.5) | 77.8 (16.1) | 80.3 (15.3) | 128.5 (16.3) | 137.1 (19.9) | 70.7 (12.7) | 75.1 (11.6) | 129.2 (7.7) | 138.0 (13.6) | 72.0 (8.1) | 77.0 (9.9) | 83.0 (15.3) | 54.2 (32.2) | 14.2 (13.5) | 42.1 (30.4) | 99.6 (1.5) | 99.1 (3.1) | 6 (3.9) | 14 (9.3) | 2 (1.3) | 1 (0.7) | 46 (29.7) | 26 (17.3) |
| Yang et al. 2022 (ENCHANTED2/MT) | 124 | 143 | 71 | 81 | 121 | 139 | 67 | 79 | NA | NA | NA | NA | NA | NA | NA | NA | NA | NA | NA | NA | NA | NA | NA | NA |

Table S3: Blood pressure montoring details of the included trials.

N., number; SD, standard deviation; NA, not available.

| **Study ID** | **Type of the used anti-hypertensive, N. (%)** | | | | | | | | | | | | | | | | | | | | | |
| --- | --- | --- | --- | --- | --- | --- | --- | --- | --- | --- | --- | --- | --- | --- | --- | --- | --- | --- | --- | --- | --- | --- |
|  | Nicardipine | | Labetalol | | Hydralazine | | Metoprolol | | Carvedilol | | Nimodipine | | Nifedipine | | Urapidil | | Sodium nitroprusside | | Nitroglycerin | | Frusemide | |
|  | Intensive | Standard | Intensive | Standard | Intensive | Standard | Intensive | Standard | Intensive | Standard | Intensive | Standard | Intensive | Standard | Intensive | Standard | Intensive | Standard | Intensive | Standard | Intensive | Standard |
| Mazighi et al. 2021 (BP-Target) | NA | NA | NA | NA | NA | NA | NA | NA | NA | NA | NA | NA | NA | NA | NA | NA | NA | NA | NA | NA | NA | NA |
| Mistry et al. 2023 (BEST-II) | 25 (86.2) | 24 (75) | 3 (10.3) | 3 (9.4) | 0 (0) | 2 (6.25) | 1 (3.4) | 2 (6.25) | 0(0) | 1 (3.125) | NA | NA | NA | NA | NA | NA | NA | NA | NA | NA | NA | NA |
| Nam et al. 2023 (The OPTIMAL BP) [ | 107 (69.0) | 28 (18.7) | 10 (6.5) | 0 (0.0) | NA | NA | NA | NA | NA | NA | NA | NA | NA | NA | NA | NA | NA | NA | NA | NA | NA | NA |
| Yang et al. 2022 (ENCHANTED2/MT) | 76 (21) | 23 (11) | 1 (0) | 1 (0) | 0 (0) | 0 (0) | 1 (0) | 0 (0) | NA | NA | 56 (15) | 33 (16) | 2 (1) | 0 (0) | 294 (80) | 145 (70) | 22 (6) | 3 (1) | 40 (11) | 10 (5) | 23 (6) | 22 (11) |

Table S4: Type of the used anti-hypertensives.

N., number; NA, not available.

| **Domain** | **Signalling question** | **Response** | **Comments** |
| --- | --- | --- | --- |
| **Bias arising from the randomization process** | 1.1 Was the allocation sequence random? | Y | Computer-generated random numbers |
|  | 1.2 Was the allocation sequence concealed until participants were enrolled and assigned to interventions? | Y |  |
|  | 1.3 Did baseline differences between intervention groups suggest a problem with the randomization process? | PN | No baseline differences |
|  | **Risk of bias judgement** | **Low** |  |
| **Bias due to deviations from intended interventions** | 2.1.Were participants aware of their assigned intervention during the trial? | Y | "Open-label" |
|  | 2.2.Were carers and people delivering the interventions aware of participants' assigned intervention during the trial? | PY |  |
|  | 2.3. If Y/PY/NI to 2.1 or 2.2: Were there deviations from the intended intervention that arose because of the experimental context? | N | Only one person of about 160 withdrew his consent so it is more likely due to reason other than the trial context. |
|  | 2.4 If Y/PY to 2.3: Were these deviations likely to have affected the outcome? | NA |  |
|  | 2.5. If Y/PY/NI to 2.4: Were these deviations from intended intervention balanced between groups? | NA |  |
|  | 2.6 Was an appropriate analysis used to estimate the effect of assignment to intervention? | Y | "Modified ITT" |
|  | 2.7 If N/PN/NI to 2.6: Was there potential for a substantial impact (on the result) of the failure to analyse participants in the group to which they were randomized? | NA |  |
|  | **Risk of bias judgement** | **Low** |  |
| **Bias due to missing outcome data** | 3.1 Were data for this outcome available for all, or nearly all, participants randomized? | Y | very small percentage of missing outcomes " 5% of missing follow-up CT scans (due to absence of or uninterpretable scans) for assessment of the primary outcome." |
|  | 3.2 If N/PN/NI to 3.1: Is there evidence that result was not biased by missing outcome data? | NA |  |
|  | 3.3 If N/PN to 3.2: Could missingness in the outcome depend on its true value? | NA |  |
|  | 3.4 If Y/PY/NI to 3.3: Is it likely that missingness in the outcome depended on its true value? | NA |  |
|  | **Risk of bias judgement** | **Low** |  |
| **Bias in measurement of the outcome** | 4.1 Was the method of measuring the outcome inappropriate? | N | it was measured by scores and adverse events |
|  | 4.2 Could measurement or ascertainment of the outcome have differed between intervention groups? | PN |  |
|  | 4.3 Were outcome assessors aware of the intervention received by study participants? | PY | "Open-label" |
|  | 4.4 If Y/PY/NI to 4.3: Could assessment of the outcome have been influenced by knowledge of intervention received? | N |  |
|  | 4.5 If Y/PY/NI to 4.4: Is it likely that assessment of the outcome was influenced by knowledge of intervention received? | NA |  |
|  | **Risk of bias judgement** | **Low** |  |
| **Bias in selection of the reported result** | 5.1 Were the data that produced this result analysed in accordance with a pre-specified analysis plan that was finalized before unblinded outcome data were available for analysis? | Y | The data in the protocol is the same as the data that reported in the paper |
|  | 5.2 ... multiple eligible outcome measurements (e.g. scales, definitions, time points) within the outcome domain? | N |  |
|  | 5.3 ... multiple eligible analyses of the data? | N |  |
|  | **Risk of bias judgement** | **Low** |  |
| **Overall bias** | **Risk of bias judgement** | **Low** |  |

Table S2: Risk of bias assessment details of Mazighi et al. 2021 (BP-Target), using ROB2.

| **Domain** | **Signalling question** | **Response** | **Comments** |
| --- | --- | --- | --- |
| **Bias arising from the randomization process** | 1.1 Was the allocation sequence random? | Y | "computer-generated permuted block randomization (block sizes 3, 6, and 9)" |
|  | 1.2 Was the allocation sequence concealed until participants were enrolled and assigned to interventions? | Y |  |
|  | 1.3 Did baseline differences between intervention groups suggest a problem with the randomization process? | N | No baseline differences |
|  | **Risk of bias judgement** | **Low** |  |
| **Bias due to deviations from intended interventions** | 2.1.Were participants aware of their assigned intervention during the trial? | Y | "Open-label" |
|  | 2.2.Were carers and people delivering the interventions aware of participants' assigned intervention during the trial? | Y |  |
|  | 2.3. If Y/PY/NI to 2.1 or 2.2: Were there deviations from the intended intervention that arose because of the experimental context? | PN | No withdrew patients |
|  | 2.4 If Y/PY to 2.3: Were these deviations likely to have affected the outcome? | NA |  |
|  | 2.5. If Y/PY/NI to 2.4: Were these deviations from intended intervention balanced between groups? | NA |  |
|  | 2.6 Was an appropriate analysis used to estimate the effect of assignment to intervention? | Y | "Full analysis data set" |
|  | 2.7 If N/PN/NI to 2.6: Was there potential for a substantial impact (on the result) of the failure to analyse participants in the group to which they were randomized? | NA |  |
|  | **Risk of bias judgement** | **Low** |  |
| **Bias due to missing outcome data** | 3.1 Were data for this outcome available for all, or nearly all, participants randomized? | Y | "Only 7 patients had missing outcome data" + "there is sensitivity analysis" |
|  | 3.2 If N/PN/NI to 3.1: Is there evidence that result was not biased by missing outcome data? | NA |  |
|  | 3.3 If N/PN to 3.2: Could missingness in the outcome depend on its true value? | NA |  |
|  | 3.4 If Y/PY/NI to 3.3: Is it likely that missingness in the outcome depended on its true value? | NA |  |
|  | **Risk of bias judgement** | **Low** |  |
| **Bias in measurement of the outcome** | 4.1 Was the method of measuring the outcome inappropriate? | PN | "It was measured by scores and adverse events " |
|  | 4.2 Could measurement or ascertainment of the outcome have differed between intervention groups? | PN |  |
|  | 4.3 Were outcome assessors aware of the intervention received by study participants? | N | "Blinded-outcome assessment" |
|  | 4.4 If Y/PY/NI to 4.3: Could assessment of the outcome have been influenced by knowledge of intervention received? | NA |  |
|  | 4.5 If Y/PY/NI to 4.4: Is it likely that assessment of the outcome was influenced by knowledge of intervention received? | NA |  |
|  | **Risk of bias judgement** | **Low** |  |
| **Bias in selection of the reported result** | 5.1 Were the data that produced this result analysed in accordance with a pre-specified analysis plan that was finalized before unblinded outcome data were available for analysis? | Y | "NCT04116112"  the data in protocol is same as the date that reported in the paper |
|  | 5.2 ... multiple eligible outcome measurements (e.g. scales, definitions, time points) within the outcome domain? | PN |  |
|  | 5.3 ... multiple eligible analyses of the data? | PN |  |
|  | **Risk of bias judgement** | **Low** |  |
| **Overall bias** | **Risk of bias judgement** | **Low** |  |

Table S3: Risk of bias assessment details of Mistry et al. 2023 (BEST-II), using ROB2.

| **Domain** | **Signalling question** | **Response** | **Comments** |
| --- | --- | --- | --- |
| **Bias arising from the randomization process** | 1.1 Was the allocation sequence random? | Y |  |
|  | 1.2 Was the allocation sequence concealed until participants were enrolled and assigned to interventions? | Y |  |
|  | 1.3 Did baseline differences between intervention groups suggest a problem with the randomization process? | N |  |
|  | **Risk of bias judgement** | **Low** | Although it was an open label study, the allocation was random, the endpoint was blinded, and there were no differences in the baseline data. |
| **Bias due to deviations from intended interventions** | 2.1.Were participants aware of their assigned intervention during the trial? | Y |  |
|  | 2.2.Were carers and people delivering the interventions aware of participants' assigned intervention during the trial? | Y |  |
|  | 2.3. If Y/PY/NI to 2.1 or 2.2: Were there deviations from the intended intervention that arose because of the experimental context? | N |  |
|  | 2.4 If Y/PY to 2.3: Were these deviations likely to have affected the outcome? | NA |  |
|  | 2.5. If Y/PY/NI to 2.4: Were these deviations from intended intervention balanced between groups? | NA |  |
|  | 2.6 Was an appropriate analysis used to estimate the effect of assignment to intervention? | Y |  |
|  | 2.7 If N/PN/NI to 2.6: Was there potential for a substantial impact (on the result) of the failure to analyse participants in the group to which they were randomized? | NA |  |
|  | **Risk of bias judgement** | **Low** | There was no deviation from the intended intervention and the appropriate analysis was used |
| **Bias due to missing outcome data** | 3.1 Were data for this outcome available for all, or nearly all, participants randomized? | Y |  |
|  | 3.2 If N/PN/NI to 3.1: Is there evidence that result was not biased by missing outcome data? | NA |  |
|  | 3.3 If N/PN to 3.2: Could missingness in the outcome depend on its true value? | NA |  |
|  | 3.4 If Y/PY/NI to 3.3: Is it likely that missingness in the outcome depended on its true value? | NA |  |
|  | **Risk of bias judgement** | **Low** | Attrition was low. |
| **Bias in measurement of the outcome** | 4.1 Was the method of measuring the outcome inappropriate? | N |  |
|  | 4.2 Could measurement or ascertainment of the outcome have differed between intervention groups? | N |  |
|  | 4.3 Were outcome assessors aware of the intervention received by study participants? | Y |  |
|  | 4.4 If Y/PY/NI to 4.3: Could assessment of the outcome have been influenced by knowledge of intervention received? | N |  |
|  | 4.5 If Y/PY/NI to 4.4: Is it likely that assessment of the outcome was influenced by knowledge of intervention received? | NA |  |
|  | **Risk of bias judgement** | **Low** | The method of measuring the outcome was appropriate and it was blind endpoint trial |
| **Bias in selection of the reported result** | 5.1 Were the data that produced this result analysed in accordance with a pre-specified analysis plan that was finalized before unblinded outcome data were available for analysis? | Y |  |
|  | 5.2 ... multiple eligible outcome measurements (e.g. scales, definitions, time points) within the outcome domain? | N |  |
|  | 5.3 ... multiple eligible analyses of the data? | N |  |
|  | **Risk of bias judgement** | **Low** | Data were analysed according to a pre specified plan. |
| **Overall bias** | **Risk of bias judgement** | **Low** | Low risk domains |

Table S4: Risk of bias assessment details of Nam et al. 2023 (The OPTIMAL BP), using ROB2.

| **Domain** | **Signalling question** | **Response** | **Comments** |
| --- | --- | --- | --- |
| **Bias arising from the randomization process** | 1.1 Was the allocation sequence random? | Y |  |
|  | 1.2 Was the allocation sequence concealed until participants were enrolled and assigned to interventions? | PY |  |
|  | 1.3 Did baseline differences between intervention groups suggest a problem with the randomization process? | N |  |
|  | **Risk of bias judgement** | **Low** | Although it was an open label trial, allocation was random and there were no baseline differences between the groups |
| **Bias due to deviations from intended interventions** | 2.1.Were participants aware of their assigned intervention during the trial? | Y |  |
|  | 2.2.Were carers and people delivering the interventions aware of participants' assigned intervention during the trial? | Y |  |
|  | 2.3. If Y/PY/NI to 2.1 or 2.2: Were there deviations from the intended intervention that arose because of the experimental context? | N |  |
|  | 2.4 If Y/PY to 2.3: Were these deviations likely to have affected the outcome? | NA |  |
|  | 2.5. If Y/PY/NI to 2.4: Were these deviations from intended intervention balanced between groups? | NA |  |
|  | 2.6 Was an appropriate analysis used to estimate the effect of assignment to intervention? | Y |  |
|  | 2.7 If N/PN/NI to 2.6: Was there potential for a substantial impact (on the result) of the failure to analyse participants in the group to which they were randomized? | NA |  |
|  | **Risk of bias judgement** | **Low** | Although it was an open label trial, there were no deviation from the intended intervention, and the appropriate analysis was used |
| **Bias due to missing outcome data** | 3.1 Were data for this outcome available for all, or nearly all, participants randomized? | Y |  |
|  | 3.2 If N/PN/NI to 3.1: Is there evidence that result was not biased by missing outcome data? | NA |  |
|  | 3.3 If N/PN to 3.2: Could missingness in the outcome depend on its true value? | NA |  |
|  | 3.4 If Y/PY/NI to 3.3: Is it likely that missingness in the outcome depended on its true value? | NA |  |
|  | **Risk of bias judgement** | **Low** | Attrition was low |
| **Bias in measurement of the outcome** | 4.1 Was the method of measuring the outcome inappropriate? | N |  |
|  | 4.2 Could measurement or ascertainment of the outcome have differed between intervention groups? | N |  |
|  | 4.3 Were outcome assessors aware of the intervention received by study participants? | Y |  |
|  | 4.4 If Y/PY/NI to 4.3: Could assessment of the outcome have been influenced by knowledge of intervention received? | N |  |
|  | 4.5 If Y/PY/NI to 4.4: Is it likely that assessment of the outcome was influenced by knowledge of intervention received? | NA |  |
|  | **Risk of bias judgement** | **Low** | The method of measuring the outcomes was appropriate, and no difference between the groups were detected |
| **Bias in selection of the reported result** | 5.1 Were the data that produced this result analysed in accordance with a pre-specified analysis plan that was finalized before unblinded outcome data were available for analysis? | Y |  |
|  | 5.2 ... multiple eligible outcome measurements (e.g. scales, definitions, time points) within the outcome domain? | N |  |
|  | 5.3 ... multiple eligible analyses of the data? | N |  |
|  | **Risk of bias judgement** | **Low** | Data were analysed according to a pre-specified analysis plan. |
| **Overall bias** | **Risk of bias judgement** | **Low** | Low risk domains |

Table S5: Risk of bias assessment details of Yang et al. 2022 (ENCHANTED2/MT), using ROB2.
